# *In Vitro* and *In Vivo* Behaviour of ^111^In Complexes of TTHA, TTHA-Bis(Butylamide) and TTHA-Bis(Glucamide): Stability, Biodistribution and Excretion Studied by Gamma Imaging

**DOI:** 10.1155/MBD.1998.259

**Published:** 1998

**Authors:** M. I. M. Prata, M. J. Ribeiro, A. C. Santos, J. A. Peters, F. Nepveu, C. F. G. C. Geraldes, J. J. P. de Lima

**Affiliations:** 1 Serviço de Biofísica e Biomatemática Fac. Medicina Univ. de Coimbra Portugal; 2 Departamento de Bioquímica e Centro de Neurociências Univ. de Coimbra Portugal; 3 Lab. of Organic Chemistry and Catalysis Delft University of Technology Delft The Netherlands; 4 Laboratoire de Synthèse Physico-Chimie et Radiobiologie Université Paul Sabatier Toulouse France

## Abstract

Aiming at radiopharmaceutical application, ^111^In_3+_ complexes of the polyaminocarboxylates TTHA, TTHA-bis(butylamide) and TTHA-bis(glucamide) were investigated. The *in vitro* stability of ^111^In(TTHA)^3−^ and ^111^In(TTHA-bis(butylamide)^-^ was evaluated by measuring the exchange of ^111^In_3+_ from the complexes to transferrin and the results were compared with those for ^111^In(DTPA)^2−^. We also performed biodistribution studies of the three ^111^In_3+_ complexes by gamma-imaging in Wistar rats and by measuring the radioactivity in their organs. TTHA and its derivatives seem to have similar *in vivo* biodistribution with prevailing renal excretion.


### *IN VITRO* AND *IN VIVO* BEHAVIOUR OF $^{111}In$ COMPLEXES OF TTHA, TTHA-BIS(BUTYLAMIDE) AND TTHA-BIS(GLUCAMIDE): STABILITY, BIODISTRIBUTION AND EXCRETION STUDIED BY GAMMA IMAGING

M. I. M. Prata[1,2], M. J. Ribeiro[1], A. C. Santos[1], J. A. Peters[3], F. Nepveu[4], C. F. G. C. Geraldes*[2] and J. J. P. de Lima[1]

[1] Serviço de Biofísica e Biomatemática, Fac. Medicina, Univ. de Coimbra,

[2] Departamento de Bioquímica e Centro de Neurociências, Univ. de Coimbra,

[3] Lab. of Organic Chemistry and Catalysis, Delft University of Technology, Delft, The Netherlands,

[4] Laboratoire de Synthèse, Physico-Chimie et Radiobiologie, Université Paul Sabatier, Toulouse, France

### Abstract

Aiming at radiopharmaceutical application, $^{111}In^{3+}$ complexes of the polyaminocarboxylates TTHA, TTHA-bis(butylamide) and TTHA-bis(glucamide) were investigated. The *in vitro* stability of $^{111}In(TTHA)^{3-}$ and $^{111}In(TTHA\text{-bis(butylamide)})^{-}$ was evaluated by measuring the exchange of $^{111}In^{3+}$ from the complexes to transferrin and the results were compared with those for $^{111}In(DTPA)^{2-}$. We also performed biodistribution studies of the three $^{111}In^{3+}$ complexes by gamma-imaging in Wistar rats and by measuring the radioactivity in their organs. TTHA and its derivatives seem to have similar *in vivo* biodistribution with prevailing renal excretion.

### 1. Introduction

Nuclides of indium have found widespread use in nuclear medicine. $^{111}In$, a $\gamma$-emitting isotope, has nearly ideal physical characteristics (it decays by electron capture emitting 2 usable gamma photons of 173 KeV and 247 KeV with a 184% photon yield [1]). $^{111}In$ has a half-life of 2.8 days which allows studies over several days with small activities administered radioactivity [2]. It also presents suitable chemical properties for *in vivo* applications: only the 3+ oxidation state occurs in water and its aqueous chemistry is dominated by its strong Lewis acidity and oxophilicity [3].

Among the chelating agents which have found applications in medicine, DTPA (diethylenetriaminepentaacetic acid) remains one of the most used [2], because it forms stable complexes with many cations and allows the preparation of bifunctional chelating derivatives; it can easily be linked to high molecular weight compounds. $^{111}In(DTPA)$ has been described as an ideal agent for scintigraphic studies of the cerebrospinal fluid pathway [4,5].

Functionalization may be important to increase the selectivity of a radiotracer and to modulate its hydrophilic/lipophilic character.

Since the biodistribution and excretion of substances injected into the blood stream are influenced by factors like molecular size, molecular weight, charge and hydrophilicity of the complex [6], we study in this work the *in vitro* and *in vivo* behaviour of $^{111}In$ complexes of the DTPA analogue TTHA (triethylenetetraaminehexaacetic acid) and of two of its derivatives, TTHA-bis(butylamide) and TTHA-bis(glucamide) (Fig.1) and compare it with the behaviour of $^{111}In(DTPA)^{2-}$. The ligands mentioned form a series of negatively charged complexes with $In^{3+}$ [7,8] with varying molecular properties.

Serum transferrin is normally only about 30% saturated with iron and retains a relatively high capacity for binding other metal ions, namely $Ga^{3+}$ and $In^{3+}$ [9]. When $In^{3+}$ is injected in the form of $^{111}InCl_3$, the metal ion is transchelated by transferrin and the radioisotope is then found in areas of high iron uptake such as bone marrow, liver and spleen [10]. Consequently, before molecules or biomolecules labelled with $^{111}In$ can be considered as valid radiotracers *in vivo*, it must be shown that the chelates are thermodynamically stable or kinetically inert towards transmetallation with transferrin.

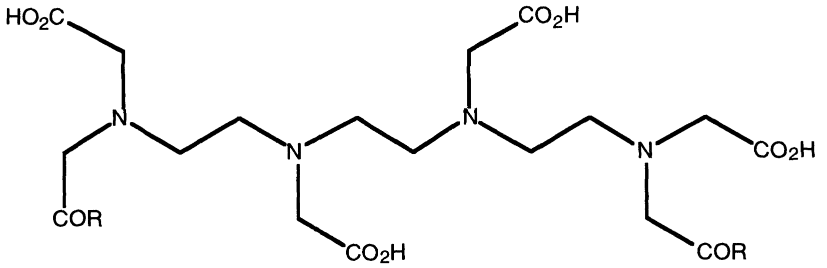

**Fig. 1.** Chemical structures of the ligands used in this study, where **R** = OH (TTHA), NH-(n-butyl) (TTHA-bis(butylamide), $TTHABuA_2$), NH-(n-glucosyl) (TTHA-bis(glucamide), $TTHAGluA_2$)

Therefore we studied the *in vitro* stability of $^{111}In(TTHA)^{3-}$ and $^{111}In[TTHA\text{-}bis(butylamide)]^{-}$ and compared it with that of $^{111}In(DTPA)^{2-}$ [11].

### 2. Materials and Methods

Reagents: $^{111}InCl_3$ , CIS-Biointernational. TTHA, Sigma. $TTHABuA_2$ and $TTHAGluA_2$ were synthesised and characterised as described elsewhere [7,8]. ITLC-SG (Instant Thin-Layer Chromatography-Silica Gel), Gelman Sciences, Inc.. Other reagents and solvents were obtained either from Aldrich or Sigma and used as supplied.

### 2.1. *In vitro* stability

The *in vitro* stability of $^{111}In(TTHA)^{3-}$ and $^{111}In(TTHABuA_2)^{-}$ was evaluated by measuring the transchelation of $^{111}In^{3+}$ to transferrin in blood serum as a function of time. This study was performed by gel filtration, after appropriate time intervals, of the solutions containing transferrin and the $^{111}In^{3+}$ complexes, using a method described in the literature [10]. A carrier free solution of 150 μl of $^{111}InCl_3$ in 0.1 N HCl (370 MBq/μg In) was mixed with 2 ml of 0.05 M sodium citrate (pH 6.5). This solution was mixed with 10 μl of a ligand solution, in such a way that a 1:1 ligand-metal ratio was obtained. A 200 μl aliquot of this mixture was added to 3 ml of human serum (final solution activity ≈ 1.5 mCi) and was subjected to gel filtration (Sephadex G-25, 1x15 cm column). The column was eluted with 0.01 M PBS buffer (pH 7.4) at a flow rate of ca. 38 ml/h. Samples were taken after a dead volume of 4 min. Activity in the samples was detected with a γ well-counter. The same procedure has been used with a transferrin solution ($2x10^{-5}$ M). Before mixing this solution with the complex, a $NaHCO_3$ solution was added (final $HCO_3^{-}$ concentration 5 mM [11]). A 200 μl aliquot of this mixture was added to 10 μl of an $^{111}In(TTHABuA_2)^{-}$ solution. At appropriate time intervals, 25 μl of this mixture was subjected to gel filtration as described above.

### 2.2. Gamma imaging

A gamma camera-computer system (GE 400 AC\STARPORT) was used for data acquisition and pre-processing. Data processing and display were performed with a CityDesk IBM AT compatible computer using software developed for these experiments.

Gamma images for the three $^{111}In^{3+}$ complexes studied in this work and for $^{111}In(DTPA)^{2-}$ as comparison, were obtained using 300-400 g Wistar rats (groups of four animals with $^{111}In(DTPA)^{2-}$and $^{111}In(TTHAGluA_2)^{-}$and groups of eight animals in the case of $^{111}In(TTHA)^{3-}$ and $^{111}In(TTHABuA_2)^{-}$. The rats were anaesthetised *via* intramuscular injection with ketamine (50 mg/ml) / chlorpromazine (2.5%) (10:3) and *ca.* 150 μCi of $^{111}In^{3+}$ complexes were injected into the femoral vein (previously catheterised with an heparinised abocat 26G) or in the tail vein. The animals were then positioned in dorsal or ventral *decubitus* over the detector. Image acquisition was initiated immediately after radiotracer injection. Sequences of 180 images (360 in the cases of $In(TTHA)^{3-}$ and $In(TTHABuA_2)^{-}$), of ten seconds each, were acquired to 64x64 matrices. Blood samples were taken during the dynamic acquisition and subsequently counted in a γ well-counter.

The efficiency of labelling of the ligands with $^{111}In^{3+}$ was checked at 24 h intervals by chromatography. This study was performed with an ITLC-SG/butanone system, analysing 10 μl of each solution of the complex. Gamma images of the chromatograms were obtained to 128×128 matrices with a total acquisition time of 15 min. For all the complexes the percentage of bound $^{111}In^{3+}$ was nearly 100%.

To analyse the transport of radiotracer over time, three regions of interest were drawn on the image files, corresponding to the thorax, liver and left kidney. From these regions, time-activity curves were obtained using home-made software.

In addition, static data were acquired at 24, 48 and 72 h after the radiotracer injection.

### 2.3. Biodistribution experiments

Two groups of four animals were injected with ca. 100 μCi of $^{111}In(DTPA)^{2-}$ and $^{111}In(TTHAGluA_2)^-$ and sacrificed 2 h later. The majors organs were removed, weighted and counted in a γ well-counter. Similar biodistribution studies were also performed with the rats used in the gamma experiments referred in the previous section sacrificed at 72 h after injection with all the $^{111}In$ chelates studied in this work.

### 3. Results and Discussion

### 3.1. *In vitro* stability

The preliminary results on the *in vitro* stability of $^{111}In(TTHA)^{3-}$ and $^{111}In(TTHABuA_2)^-$ in blood serum and in a transferrin solution are summarised in Table 1. Literature data on $^{111}In(DTPA)^{2-}$ [12] are included for comparison. The presently studied complexes dissociate more rapidly than $^{111}In(DTPA)^{2-}$. This is in agreement with published thermodynamic stability data for the ligands and for the transferrin complexes [pM (In(tf)) = 20.4, pM $(In(TTHA)^{3-})$ = 22.88, pM $(In(TTHABuA_2)^-)$ =19.43 and pM $(In(DTPA)^{2-})$ = 24.72] [7,13], which reflect the structures of the chelates in solution [7,14].

**Table 1.** Time dependence of the percent dissociation of the $^{111}In$-chelates in blood serum and in a transferrin (tf) solution.

| Time(h) | % Dissociation | | | |
|---|---|---|---|---|
| | TTHA blood serum | $TTHABuA_2$ | | DTPA blood serun |
| | | Blood serum | tf solution | |
| 2 | 0.30 | 1.64 | 0.37 | — |
| 24 | 2.36 | — | 7.54 | 1.5 [12] |
| 48 | 5.43 | 3.26 | — | <3.0 [12] |

### 3.2. Images and biodistribution data

Fig.2 represents the averaged time-activity curves, obtained from the dynamic acquisitions for each region of interest. The curves were smoothed and normalised for the maximum activity of each one. The complexes studied undergo an early retention, both in kidneys and in liver and spleen. These results contrast with the time-activity curves obtained for the $^{111}In(DTPA)^{2-}$ complex, where the liver-spleen curve is similar to the thorax curve, corresponding to blood activity. The thorax curves for the $^{111}In(TTHA)^{2-}$, $^{111}In(TTHABuA_2)^-$ and $^{111}In(TTHAGluA_2)^-$ chelates also correspond only to blood activity.

The scintigraphic images at 30 minutes, 24 h and 72 h after $^{111}In$ chelates injection in rats are illustrated in Fig.3. In the early images, and for all the complexes, the activity is preferentially localised in kidneys. In some of these images the injection site is also observed. After 24 h the activity was spread out and was then localised mainly in the abdominal region and in the kidneys. The 72 h images show the same behaviour, but it is particularly noticeable that $^{111}In(TTHAGluA_2)^-$ has higher uptake by the liver-spleen region.

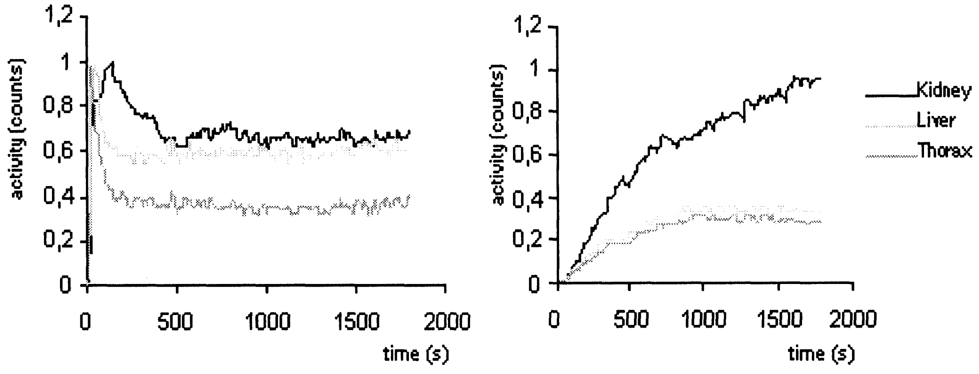

(a) (b)

**Fig. 2.** Time-activity curves for **a)** $^{111}In(TTHABuA_2)^-$ **b)** $^{111}In(DTPA)^{2-}$

The biodistribution results (in percent of injected dose per gram of organ) obtained at 2 h and 72 h are shown in Fig. 4 and agree with the gamma-imaging. It can be noticed that in contrast to $^{111}In(DTPA)^{2-}$, $^{111}In(TTHAGluA_2)^-$ has low tissue specificity and undergoes both renal and hepatobiliary clearance. The biodistribution results obtained at 72 h (Fig.4) show that for all the complexes the activity is preferentially localised in the kidneys. This indicates renal excretion of the chelates, which is consistent with their structure, molecular weight and hydrophilicity.

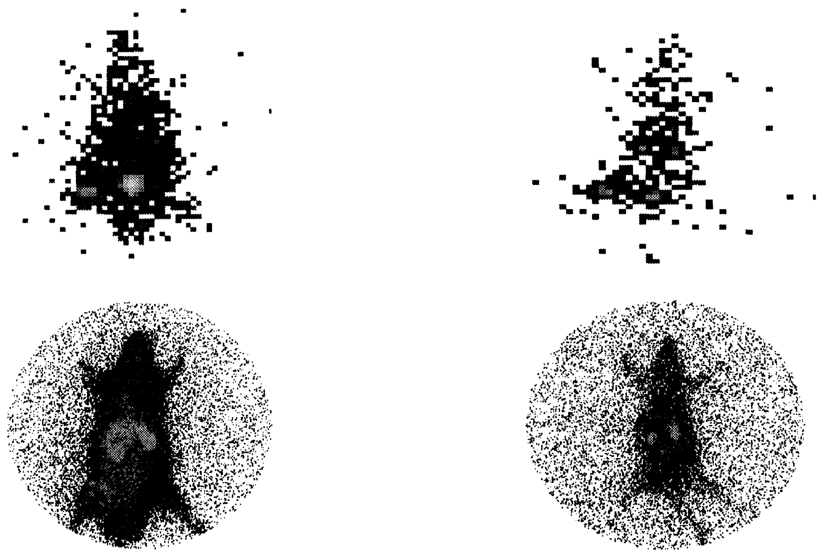

(a) (b)

**Fig. 3.** Scintigraphic images at 30 min and 48 h (from top to bottom) after injection with **a)** $^{111}In(TTHA)^{3-}$ and **b)** $^{111}In(TTHAGluA_2)^-$

The high late retention of the $^{111}$In complexes of TTHA, $TTHABuA_2$ and $TTHAGluA_2$ by the reticulo-endothelial system may be related with the formation of colloidal particles of indium hydroxide associated with partial demetallation of the chelates [15], in addition to the natural occurrence of the complexes. High radioactivity levels in blood after 30 minutes suggest that the complexes may bind to serum proteins, perhaps albumin, but further *in vitro* studies are necessary to validate this hypothesis.

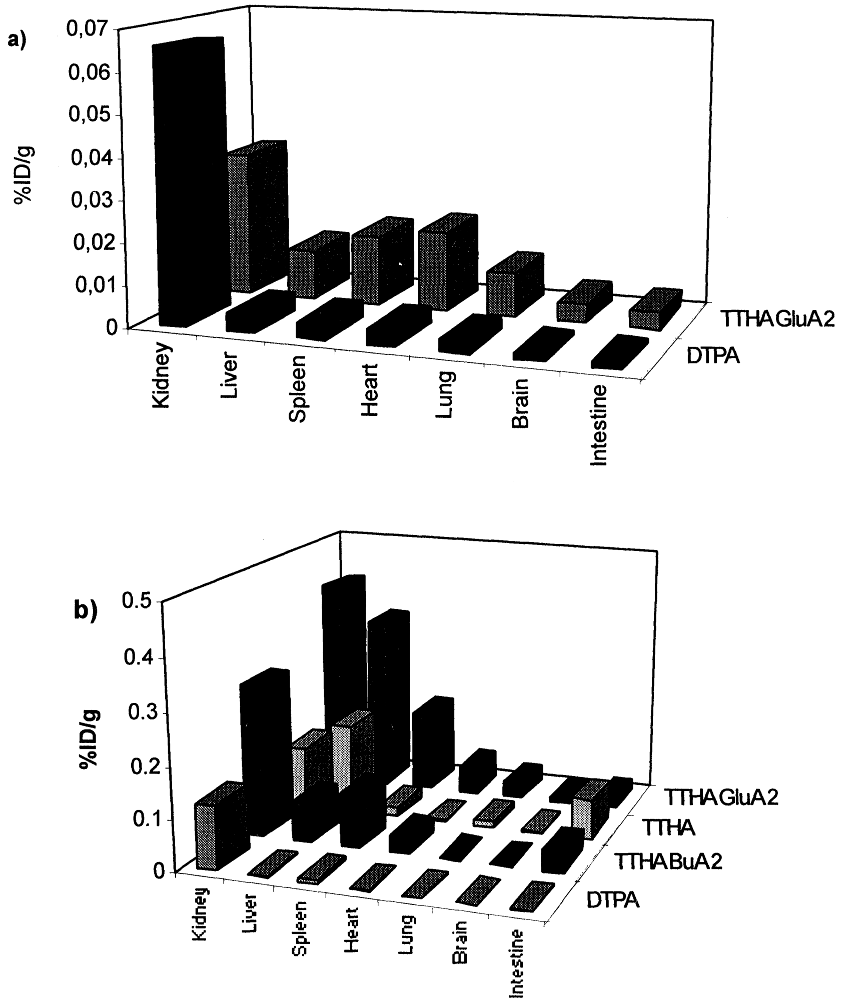

**Fig. 4** Biodistribution of $^{111}In$ metal complexes in rat tissues at **a)** 2 h and **b)** 72 h after injection of the chelates.

None of the complexes passes through the blood-brain barrier, as expected for high molecular weight and non-lipophilic complexes. There is no evidence of bone marrow accumulation, which is seen when the indium-transferrin complex is formed [16].

In conclusion TTHA and its derivatives have similar *in vivo* behaviour and the linkage of the lipophilic side chains in the case of $TTHABuA_2$ and $TTHAGluA_2$ does not seem to influence the biodistribution and clearance of these complexes.

**Acknowledgements.** The authors thank the financial support from the Fundação para a Ciência e Tecnologia (FCT) (Praxis XXI project 2/2.2/SAU/1194/95), the BIOMED II (MACE Project), COST Chemistry D8 Program of the European Union and l'Association pour la Recherche contre le Cancer (ARC).

### References

[1] Holde N. E, "Table of the Isotopes ", *Handbook of Chemistry and Physics, NewYork* (1990).
[2] Jurisson S., Berning D., Jia W., Ma D., *Chem. Rev*, **93** (1993) 1137.
[3] Hancock R. D., Martell A. E., *Chem. Rev*, **89** (1989) 1875.
[4] Goodwin D. A., Song C. H. Finston R. and Martin P., *Radiology*, **108** (1973) 91.
[5] Chilton H. M., Cowan R. J. *Pharmaceuticals in Medical Imaging*, Swanson D. P., Chilton H. M., Thrall J. H., Eds, Macmillan Publishing Co., New York (1990) 621.

[6] Lauffer R. B., *Chem. Rev.,* **87** (1987) 901.

[7] Achour B.,Costa J. Delgado R., Garrigues E., Geraldes C. F. G. C., Korber N., Nepveu F. and Prata M. I., *Inorg. Chem.,* in press, and references therein.

[8] Zitha-Bovens E., Laurent S. VanderElst L., Muller R.N., Bekkum H. van, and Peters J.A., to be published

[9] J. C. Cannon and N. D. Chasteen, *Biochemistry*, **14** (1975) 4573.

[10] Ruser G., Ritter W., Maecke H., *Bioconjugate Chem.*, **1** (1990) 345.

[11] Harris W. R., Pecoraro V. L., *Biochemistry,* **22** (1983) 292.

[12] Riesen A., Kaden T. A., Ritter W., Maecke H., *J. Chem. Soc. Chem. Comm.*, (1989) 460.

[13] Taliaferro C. H., Motekaitis R. J. and Martell A. E., *Inorg. Chem.*, **23** (1984) 1188.

[14] Maecke H. R., Riesen A. and Ritter W., *J. Nuclear Medicine,* **30** (1989) 1235.

[15] Subramanian K. M., Wolf M., *J. Nuclear Medicine,* **31** (1990) 1084.

[16] Goodwin D. A.,Goode R.,Brown L.,Imbormone C. J., *Radiology,* **100** (1971) 175.

**Received: April 30, 1998 - Accepted: May 11, 1998 - Received in revised camera-ready format: May 12, 1998**

